# Genomic and Secondary Metabolite Analyses of *Streptomyces* sp. 2AW Provide Insight into the Evolution of the Cycloheximide Pathway

**DOI:** 10.3389/fmicb.2016.00573

**Published:** 2016-05-03

**Authors:** Elizabeth R. Stulberg, Gabriel L. Lozano, Jesse B. Morin, Hyunjun Park, Ezra G. Baraban, Christine Mlot, Christopher Heffelfinger, Gillian M. Phillips, Jason S. Rush, Andrew J. Phillips, Nichole A. Broderick, Michael G. Thomas, Eric V. Stabb, Jo Handelsman

**Affiliations:** ^1^Department of Molecular, Cellular and Developmental Biology, Yale UniversityNew Haven, CT, USA; ^2^Department of Bacteriology, University of Wisconsin-MadisonMadison, WI, USA; ^3^Department of Chemistry, Yale UniversityNew Haven, CT, USA; ^4^Department of Microbiology, University of GeorgiaAthens, GA, USA

**Keywords:** glutaramide antibiotics, cycloheximide, neutramycin, hygromycin A, natural product biosynthesis, bioinformatics

## Abstract

The dearth of new antibiotics in the face of widespread antimicrobial resistance makes developing innovative strategies for discovering new antibiotics critical for the future management of infectious disease. Understanding the genetics and evolution of antibiotic producers will help guide the discovery and bioengineering of novel antibiotics. We discovered an isolate in Alaskan boreal forest soil that had broad antimicrobial activity. We elucidated the corresponding antimicrobial natural products and sequenced the genome of this isolate, designated *Streptomyces* sp. 2AW. This strain illustrates the chemical virtuosity typical of the *Streptomyces* genus, producing cycloheximide as well as two other biosynthetically unrelated antibiotics, neutramycin, and hygromycin A. Combining bioinformatic and chemical analyses, we identified the gene clusters responsible for antibiotic production. Interestingly, 2AW appears dissimilar from other cycloheximide producers in that the gene encoding the polyketide synthase resides on a separate part of the chromosome from the genes responsible for tailoring cycloheximide-specific modifications. This gene arrangement and our phylogenetic analyses of the gene products suggest that 2AW holds an evolutionarily ancestral lineage of the cycloheximide pathway. Our analyses support the hypothesis that the 2AW glutaramide gene cluster is basal to the lineage wherein cycloheximide production diverged from other glutarimide antibiotics. This study illustrates the power of combining modern biochemical and genomic analyses to gain insight into the evolution of antibiotic-producing microorganisms.

## Introduction

The Actinobacteria comprise a large bacterial phylum commonly found in soil and aquatic environments. Within the Actinobacteria, the genus *Streptomyces* has been a source of diverse and clinically important bioactive metabolites, including the antibiotics streptomycin and tetracycline, the antifungal amphotericin B, the antihelminthic avermectin, the antitumor mitomycin C, and the immunosuppressants rapamycin and tacrolimus (FK506). *Streptomyces* genomes are typically large (8–10 Mb), extremely G+C-rich, and often encode multiple biosynthetic gene clusters for secondary metabolites. For example, up to twenty-five such clusters were identified in the genome of *Streptomyces avermitilis* (Ikeda et al., 2003). These metabolites often work synergistically, as in the case of cephamycin C, a beta-lactam antibiotic, and clavulanic acid, a beta-lactamase inhibitor produced by *Streptomyces clavuligerus* (Jensen and Paradkar, 1999). These secondary metabolites are not essential for bacterial growth but instead have important ecological roles in mediating microbe–microbe and bacteria–host interactions. There are several mutualistic interactions reported where diverse invertebrates and plants guard and feed different *Streptomyces* strains, with the symbionts apparently producing various secondary metabolites to protect their host from pathogenic microorganisms (Seipke et al., 2012). Humankind has exploited these secondary metabolites, with the use of antibiotics being one of the greatest medical advances of the 20th century.

Given the increasing number of multidrug-resistant bacteria and the threat that antibiotics in current use will lose efficacy toward many pathogens, further characterization of *Streptomyces* spp. and other Actinobacteria is important to help fill the critical need for new antibiotics. Despite being tapped as antibiotic sources for decades, it is estimated that *Streptomyces* spp. potentially produce up to 100,000 antimicrobial metabolites, of which only a small proportion have been identified (Watve et al., 2001). Understanding how the Actinobacteria evolved such a diverse chemical arsenal will further enable strategies to engineer new, or as yet undiscovered, metabolic pathways and may translate to the design and production of new antibiotics *in vitro*.

The chemical complexity of known Actinobacterial secondary metabolites is reflected in similarly complex genetic underpinnings. For example, *Saccharopolyspora erythraea* expresses 20 genes to produce the macrolide antibiotic erythromycin (Staunton and Wilkinson, 1997). The genes for particular secondary metabolite biosynthetic pathways tend to be clustered, a feature thought to permit their co-regulation and spread by horizontal gene transfer. On the other hand, new chemistries can arise through distinct genetic pathways, which may be unlinked originally, but later join together in the same genome. Accordingly, the discovery of new chemical scaffolds or modifications to known molecules will often dovetail with the elucidation of their genetic evolution. Such analyses will be aided by the increasing number of complete or draft bacterial genomes available, including many for *Streptomyces* species. This genomic database creates a unique opportunity to identify diverse biosynthetic gene clusters and understand how these secondary metabolite pathways evolve, diversify, and generate the high chemical diversity present in *Streptomyces*.

Here, we report the characterization of *Streptomyces* sp. 2AW, an Alaskan soil isolate that produces at least three structurally unrelated antimicrobial metabolites – hygromycin A, neutramycin, and cycloheximide. By using large amounts of high-quality genomic DNA, which reduced the need for extensive PCR amplification, we were able to overcome the challenges inherent in sequencing a large, G+C-rich genome. We used sequence alignment and protein prediction algorithms to identify the putative biosynthetic gene clusters for the three antimicrobial metabolites. Unexpectedly, we found that the genes of the cycloheximide biosynthetic pathway of *Streptomyces* sp. 2AW are separated into two independent clusters in the genome. Based on phylogenetic analysis and data mining of several cycloheximide-like gene clusters in the genomes of other *Streptomyces* species, we propose an evolutionary framework for cycloheximide and other glutarimide metabolites in which the gene clusters diversified from a conserved polyketide synthase with five modules.

## Materials and Methods

### Culture Conditions

*Streptomyces* sp. strain 2AW was collected from boreal forest soil in Alaska as described elsewhere (Schloss et al., 2010) and then cultured on oatmeal agar plates (60 g oatmeal and 12.5 g agar per liter water, mixed in a blender until mostly smooth, pH adjusted to 6.0 with NaOH or HCl, autoclaved 45 min at 120°C, shaken, then poured into 100-mm Petri dishes). Cultures were grown at 28°C for 2 weeks or until covered with gray spores. Spores were collected by flooding plates with water and rubbing off the spores using sterile plastic loops. Spore suspensions were then vortexed, briefly sonicated, and stored at −80°C. *Pythium ultimum* was a gift from the laboratory of Scott Strobel (Yale University). It was cultured on the benchtop on potato dextrose media (Difco Laboratories, Detroit).

### Bioassays for Antimicrobial Activity

For measuring antibacterial activity, approximately 1–5 mg of each fraction (dry weight) was spotted on Luria-Bertani agar (LBA). A 120 μL overnight culture of *Escherichia coli* or *Bacillus subtilis* was mixed with 12 mL of soft LBA (0.8% agar) and poured over each plate. Plates were incubated overnight at 28°C and then inspected for zones of inhibition. Eukaryotic inhibition was assayed by spotting one side of a potato dextrose or International *Streptomyces* Project Media #2 (ISP2) plates with each fraction, as above, and adding a plug of *Pythium*-containing potato dextrose agar to the other (Shirling and Gottlieb, 1966). Plugs were taken from plates after 3–6 days of *Pythium* growth using autoclaved sections of plastic drinking straws. Inhibition was determined by zones of reduced growth of *Pythium* after 2 days at room temperature.

### Chromatography

Compounds were purified using a Biotage Isolera One system with a 12-g SiliCycle C18 17% cartridge, 230- to 400-μm particle size, and a 40- to 63-μm mesh or the Shimadzu LC-20AT high pressure liquid chromatograph with a Restek Ultra II C18 reversed-phase column (150 mm length, 4.6-mm i.d., 5.0-μm particle size, and 100-Å pore size). Liquid-chromatography/mass spectrometry was performed on a Waters Synapt G1 Q-TOF mass spectrometer coupled to a Waters Acquity UPLC. Data were routinely collected under positive electrospray conditions.

### Small Molecule Purification

For extraction of *Streptomyces* metabolites from solid cultures, cultures were grown on ISP2. Agar was removed from culture plates, placed in glass vessels, frozen at −80°C for 1–3 h, defrosted at room temperature for 1 h, and submerged in methanol. The glass vessels were shaken at room temperature for 8 h, and agar was filtered from the crude extract with a sintered-glass funnel and under vacuum.

For purification of metabolites from solid culture, crude *Streptomyces* sp. 2AW extract was obtained from 90 ISP2 plates (30 mL each) as described above. The extract was concentrated using a rotary evaporator. Approximately 0.75 g of crude extract was resuspended in 1 mL of deionized water and purified using the Biotage Isolera system using an RP-C18 cartridge. The full gradient ran from 5% acetonitrile increasing to 15% over 25 min, then to 40% over 11 min, then to 100% over 2 min and held at 100% for 4 min at a flow rate of 12 mL/min. Fractions that eluted with 30% acetonitrile were found to contain cycloheximide, which was determined to be the active component against *P. ultimum* in extracts of *Streptomyces* sp. 2AW. Fractions on either side of the cycloheximide-containing fraction exhibited a range of antibacterial activity, but they had no activity against *P. ultimum.* The amounts of these components isolated from this initial column were too low, and the they were not pure enough, for structure determination. Scale-up of the culturing and purification process for *Streptomyces* sp. 2AW extracts involved fractionation using multiple runs of reversed-phase C18 chromatography by either HPLC or the Biotage Isolera system. Fractions were pooled following identification based on activity against either both Gram-positive (*B. cereus*) and Gram-negative (*E. coli*) or Gram-positive only, as determined using overlay assays. The Biotage gradient was as follows: 5% acetonitrile increasing to 15% over 25 min, then to 40% over 11 min, then to 100% over 2 min and held at 100% for 4 min at a flow-rate of 12 mL/min. The material active against both Gram-positive and Gram-negative bacteria eluted between 9 and 21 min; the material active only against Gram-positive bacteria eluted between 25 and 41 min. The HPLC gradient was as above; the material active against both Gram-positive and Gram-negative bacteria eluted between 19 and 21 min, and the material active against Gram-positive bacteria only eluted only between 34 and 38 min.

These compounds were obtained from a total of 3.2 L of *Streptomyces* sp. 2AW culture grown on ISP2 agar and extracted as described above. The combined active fractions were purified further by reversed-phase HPLC. Yields for the purified materials were: 7 mg of the compound active against both Gram-positive and Gram-negative bacteria (hygromycin A and/or epi-hygromycin A, and methoxyhygromycin A), and 3 mg of neutramycin, the compound active against only Gram-positive bacteria.

### Elucidation of Antibiotic Structures

Nuclear magnetic resonance (NMR) spectra were recorded in D_2_O, CD_3_OD, or CDCl_3_ at 25°C using Bruker Avance 500 MHz, Varian Inova 500 MHz, and Agilent 600 MHz (3 mm cold probe) instruments, as indicated on the spectra in the **Supplementary Figures S1–S4** and **Supplementary Tables S1–S3**. Spectra run in D_2_O were not re-referenced. Spectra run in CD_3_OD were referenced to the residual solvent peaks at 3.31 ppm and 49.0 ppm for ^1^H and ^13^C NMR, respectively. Spectra run in CDCl_3_ were referenced to residual solvent peaks at 7.25 and 77.0 ppm for for ^1^H and ^13^C NMR, respectively. Accurate-mass MS data were recorded on a Waters Synapt G1 mass spectrometer after calibration with a sodium formate solution with a mass range from 100 to 2000 m/z. System suitability was obtained during each run, in which the difference between the calculated and observed m/z values were less than 5 ppm for each of four standard compounds, with m/z ranging from 152 to 609.

### DNA Sequence Analysis

Initial sequencing of *Streptomyces* sp. 2AW was conducted by the J. Craig Venter Institute using a GS FLX platform. Further library construction and sequencing were performed by the Yale Center for Genome Analysis^1^. One μg of high quality genomic DNA was sonically sheared to an average fragment size of 1 kb (Covaris). gDNA was obtained using a standard genomic phenol–chloroform extraction (Kieser et al., 2000), purified using magnetic AMPure XP beads (Beckman Coulter), selectively precipitated by mass and re-bound to the beads using a 20% polyethylene glycol, 2.5-M NaCl solution. The fragments were given blunt, phosphorylated ends with T4 DNA polymerase and T4 polynucleotide kinase. A single adenine residue was added to the 3′ end of each fragment, and custom adapters (IDT) were ligated to each fragment using T4 DNA ligase. The library was run on a 2% pre-cast gel (Invitrogen) and ∼1-kb inserts were excised, subjected to three cycles of PCR amplification using custom-made primers (IDT) containing unique 6-bp indices. The library was sequenced on an Illumina MiSeq using 2 bp × 250 bp reads.

454 paired-end reads were assembled using Newbler software^2^ with default settings, generating 16 scaffolds. Illumina reads were first trimmed using Trimmomatic (Bolger et al., 2014), using the follow setting LEADING:30 TRAILING:30 SLIDINGWINDOW:7:30 MINLEN:2. Gaps in the scaffolds were closed using the trimmed Illumina reads by GapFiller (Boetzer and Pirovano, 2012) with the follow settings: bwa algorithm; 722, average insert length; 0.30, insert length expected error; 20, m; 2, o; 5, t; 0.7, r; 15, n; 500, d; 3, g; 5, T; 4, i. Gaps were closed in the biosynthetic pathway genes with PCR and standard Sanger sequencing performed by the DNA Analysis Facility on Science Hill at Yale.

Genomic regions containing antibiotic biosynthetic gene clusters were identified using AntiSMASH software (Weber et al., 2015), IMG-AB database prediction (Hadjithomas et al., 2015) and BLAST alignment tools (Altschul et al., 1990) to align *Streptomyces* sp. 2AW sequences to related clusters. *Streptomyces* sp. 2AW genome alignments were visualized using Circos software (Krzywinski et al., 2009).

### Phylogenetic Analysis

Protein sequence alignments were performed with MAFFT version 7 (Katoh and Standley, 2013) and were manually adjusted using as a guide the residues-wise confidence scores generated by GUIDANCE2 (Sela et al., 2015). ProtTest 3 was used to select the best-fit model of amino acid replacement (Darriba et al., 2011). Phylogenetic relationships were inferred by maximum likelihood using RAxML-HPC2 on XSEDE conducted on the CIPRES project (Miller et al., 2010) cluster at the San Diego Supercomputer Center. Phylogenetic trees were visualized using FigTree software^3^.

## Results and Discussion

### Identification of Antibiotics

**FIGURE 1:**
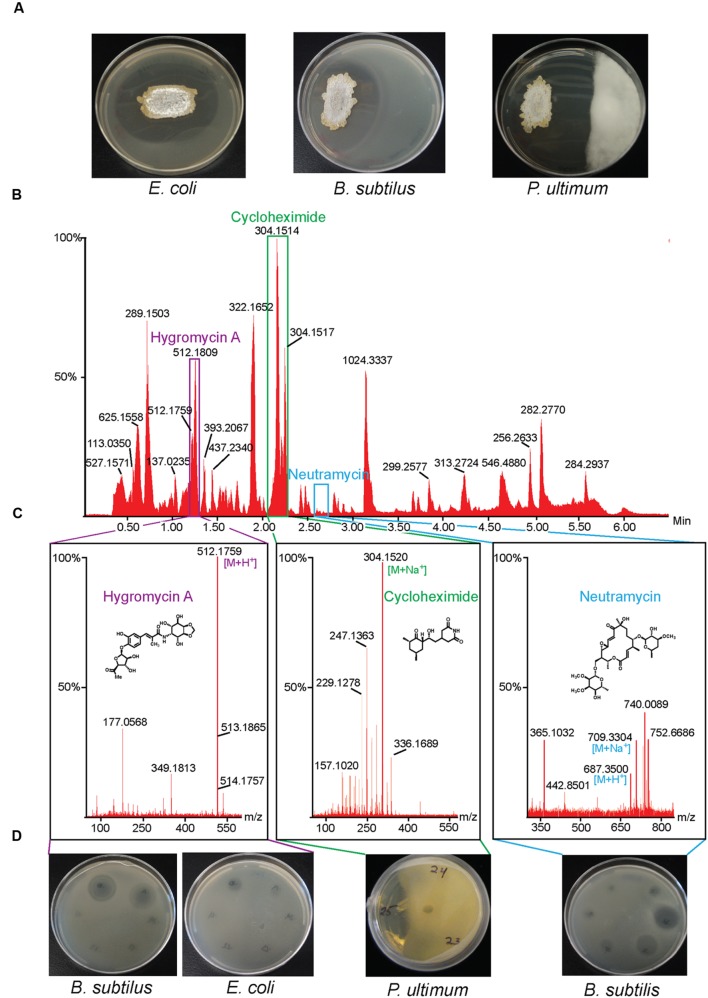
**Isolation of antibiotics from *Streptomyces* sp. 2AW.** **(A)** *Streptomyce*s sp. 2AW grown on solid culture with Gram-positive bacterium *Bacillus subtilis*, the Gram-negative bacterium *Escherichia coli*, and the eukaryotic pathogen *Pythium ultimum*. **(B)** UPLC chromatogram of the *Streptomyces* sp. 2AW extract, and **(C)** mass spectra of each active component confirming the exact masses and its corresponding structure. **(D)** Zones of inhibition of the HPLC fractions showing the fractions’ activity against *B. subtilis*, *E. coli*, and *P. ultimum*.

We isolated a large collection of diverse bacteria from the Bonanza Creek Long-Term Ecological Research Area in Alaska and screened the collection for inhibitory activity toward *B. subtilis* (Schloss et al., 2010). We identified *Streptomyces* sp. strain 2AW, which was subsequently also shown to inhibit *E. coli* and the oomycete plant pathogen, *P. ultimum.* We purified three compounds by bioassay-guided fractionation, one active against *P. ultimum*, one against *B. subtilis*, and one against both *E. coli* and *B. subtilis*. The structures of these three compounds, obtained by NMR spectroscopy and/or mass spectrometry (MS), are consistent with cycloheximide, neutramycin, and hygromycin A, respectively (**Figure 1**).

Cycloheximide is an antifungal antibiotic first discovered in *Streptomyces griseus* (Lyons and Pridham, 1966). It belongs to the glutarimide class of antibiotics, all of which contain a glutarimide ring with different polyketide substituents (Obrig et al., 1971). Cycloheximide inhibits the eukaryotic ribosome by binding to the E-site and interfering with the subsequent binding of deacylated tRNA to this site (Schneider-Poetsch et al., 2010).

Neutramycin is a neutral macrolide first identified from *S. rimosus* and *S. luteoverticillatus* with inhibitory activity against Gram-positive bacteria (Lefemine et al., 1963; Graziani et al., 2003). The lack of a C6-methyl group differentiates neutramycin from the related antibiotic chalcomycin (**Supplementary Figure S5B**) (Mitscher and Kunstmann, 1969; Ward et al., 2004). Dihydrochalcomycin, an antibiotic isolated from *Streptomyces* sp. KCTC 0041BP, is also structurally similar to neutramycin, lacking a C6-methyl group and a Δ^10,11^ double bond (Pageni et al., 2010).

The bioactivity, and accurate mass, and mass spectral fragmentation patterns of the third antibiotic were consistent with the glycosylated polyketide hygromycin A or epihygromycin A (Wakisaka et al., 1980). NMR spectroscopy and MS also revealed a compound whose structure we have been unable to elucidate that might be related to hygromycin A; we also found a previously characterized hygromycin A biosynthetic intermediate methoxyhygromycin A (**Supplementary Figure S5**) (Yoshida et al., 2014). Hygromycin A was first discovered in isolates of *S. hygroscopicus* (before the discovery in the same species of the structurally unrelated, but well-known hygromycin B). Hygromycin A inhibits the bacterial ribosomal peptidyltransferase in both Gram-negative and Gram-positive bacteria (Guerrero and Modolell, 1980).

### Sequence of *Streptomyces* sp. 2AW Genome

The *Streptomyces* sp. 2AW genome was sequenced by 454 and Illumina methodologies, resulting in 36 contigs organized into 12 scaffolds. The total length of the assembly was 9,449,583 bp with an average G+C content of 70%. **Supplementary Table S4** summarizes the basic genomic characteristics. We used Prodigal software (Hyatt et al., 2010) coupled with the IMG/ER pipeline (Markowitz et al., 2009) to identify 8,241 putative protein-encoding genes, 70 tRNA genes, and one rRNA operon. **Supplementary Table S5** lists the genes identified along with its corresponding annotation. As one assessment of genome comprehensiveness, we evaluated the completeness of a particular biochemical pathway (the pyrimidine biosynthetic pathway), which did appear complete in our database (**Supplementary Figure S6**). We detected 28 putative secondary metabolite gene clusters from a manual comparison of the AntiSMASH software prediction and the IMG-AB database prediction (**Supplementary Figure S7**) (Hadjithomas et al., 2015; Weber et al., 2015). The genome is available at JGI Genome Portal^4^ with the IMG Object ID 2606217189.

### Identification of Biosynthetic Clusters

#### Hygromycin A

Analysis of the *Streptomyces* sp. 2AW genome revealed a region that contains 78% nucleotide identity to the published hygromycin A biosynthetic genes *hyg2* to *hyg27* from *S. hygroscopicus* strain NRRL 2388 (Palaniappan et al., 2006). We predict that these genes encode the hygromycin A biosynthetic enzymes, and based on the structural similarities between hygromycin A, methoxyhygromycin A, and the hygromycin A derivative, these genes may encode enzymes that catalyze modifications of the same compounds (**Supplementary Figure S8A**).

#### Neutramycin

We found a gene cluster in 2AW that is likely to be responsible for the biosynthesis of neutramycin based on sequence identity and similar genetic organization of the gene clusters underlying production of the related antibiotics chalcomycin and dihydrochalcomycin in *S. bikiniensis* and *Streptomyces* sp. KCTC 0041BP, respectively (**Supplementary Figure S8B**) (Ward et al., 2004; Pageni et al., 2010).

#### Cycloheximide

**FIGURE 2:**
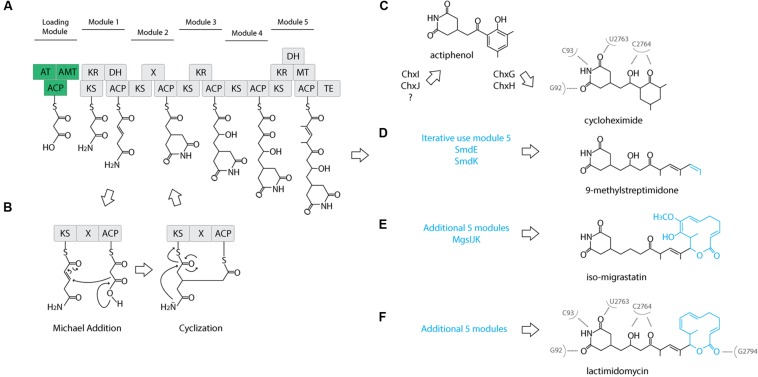
**Proposed cycloheximide and other glutaramide biosynthetic pathways.** **(A)** Proposed biosynthetic pathway of the five conserved modules of the AT-less PKS of glutaramide antibiotics. A loading module (green): ACP, acyl carrier protein; AT, malonyl-CoA ACP transacylase; AMT, amidotransferase. An AT-less modular type I polyketide synthase (PKS) region (gray): KS, ketosynthase; DH, dehydratase; ER, enoylreductase; MT, methyltransferase; ACP, acyl carrier protein; X, uncharacterized cyclization domain; TE, thiosesterase. **(B)** Proposed catalytic domains and biosynthetic pathway including the Michael addition catalyzed by the X uncharacterized domain to generate the characteristic glutaramide ring. **(C)** Proposed pathway for cycloheximide formation mediated by tailoring gene cluster. **(D)** Proposed pathway for 9-methylstreptomidone formation. **(E)** Proposed pathway for iso-migrastatin formation. **(F)** Proposed pathway for lactimidomycin formation. **(C,E)** Nucleotides of *Saccharomyces cerevisiae* 25S rRNA that directly interact with cycloheximide and lactimidomycin in the ribosome.

The cycloheximide biosynthetic gene cluster and proposed biosynthetic pathway was recently identified from *Streptomyces* sp. YIM56141 (Yin et al., 2014). This gene cluster shares similar gene architecture with other glutarimide antibiotic gene clusters (Lim et al., 2009; Wang et al., 2013) including: an acyl carrier protein (ACP), a malonyl-CoA ACP transacylase (AT), an asparagine synthase or amidotransferase (AMT), and an AT-less modular type I polyketide synthase (PKS). The first three genes are proposed to form the loading module, where AMT installs the amide nitrogen at the free carboxylate, and coupled with the first two modules of the PKS, they produce the characteristic glutarimide ring (**Figure 2A**). In the second elongation module, it was proposed that inclusion of an uncharacterized “X” domain catalyzes the cyclization of the glutarimide ring in 9-methylstreptimidone (Wang et al., 2013). The AT loads the ACP with a malonate, but instead of typical decarboxylative addition to the thioester of the ketosynthase-bound molecule, elongation occurs through a Michael addition. This introduces a branch point and leads to an intermediate that is tethered to both the ACP and the KS. The glutarimide ring is formed through the intramolecular acylation of the amide nitrogen by the thioester, resulting in the release of the molecule from the KS (**Figure 2B**). A cycloheximide-specific tailoring gene cluster downstream of the PKS-encoding genes participates in the modification of the nascent polyketide intermediate, first to actiphenol and finally to cycloheximide (**Figure 2C**).

**FIGURE 3:**
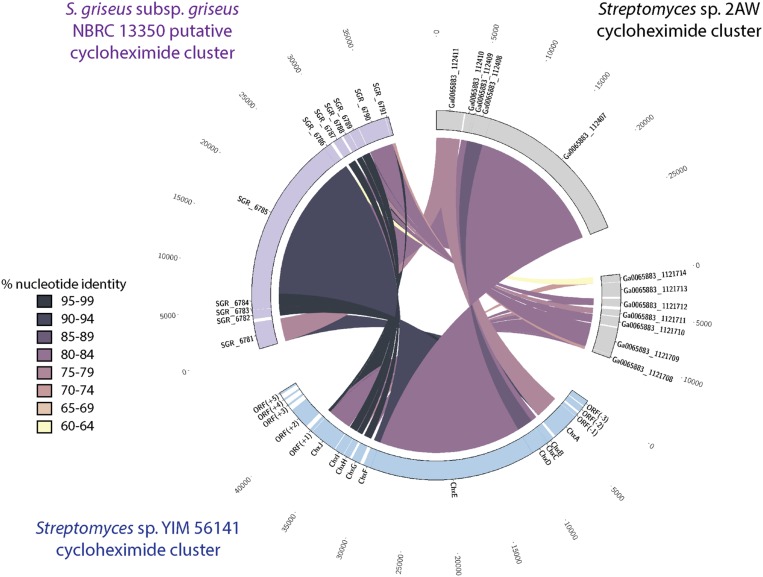
**Comparison of the cycloheximide biosynthetic cluster from *Streptomyces* sp. 2AW to the putative clusters *Streptomyces* sp. YIM 56141 and *S. griseus* subsp. *griseus* NBRC 13350.** Putative *Streptomyces* sp. 2AW cycloheximide genes homologous to *Streptomyces* sp. YIM 56141 genes *chxABCDE* are separated from those homologous to *chxFGHIJ*.

We identified a similar gene cluster in the *Streptomyces* sp. 2AW genome. However, the tailoring gene cluster in strain 2AW has an additional gene, and the entire tailoring cluster is located in a different part of the genome, not adjacent to the core PKS biosynthetic gene cluster as in YIM56141 (**Supplementary Figure S7**). To further explore this unexpected genetic arrangement, we identified a cycloheximide biosynthetic gene cluster in the genome of *S. griseus* subsp. *griseus* NBRC13350 with the core PKS biosynthetic genes adjacent to the tailoring gene cluster that appeared more similar to YIM56141 (**Figure 3**).

### Proposed Evolutionary Origin of the Cycloheximide Biosynthetic Gene Cluster

Based on the relatively low similarity between the cycloheximide gene cluster in 2AW and the other strains, along with the separation of the tailoring gene cluster in 2AW, we hypothesized that the 2AW cycloheximide biosynthetic genes may reflect a more ancestral version of the cycloheximide biosynthetic pathway. In this model, the tailoring gene cluster initially evolved in a different part of the chromosome, or in another genome, from the glutarimide core PKS gene cluster, and an *in trans* interaction of these two gene clusters gave rise to cycloheximide as a novel secondary metabolite. Subsequent evolution of the pathway resulted in the gene arrangement found in cycloheximide producers YIM56141 and NBRC13350, with the core PKS and cycloheximide-specific genes clustered together.

We tested this hypothesis by comparing the proteins that encode the tailoring gene clusters of the different cycloheximide biosynthetic pathways, including a transcriptional regulator, *chxF*; a enoylreductase, *chxG*; a ketoreductase, *chxH*; a cytochrome P450 oxidoreductase, *chxI*; and a three-domain carboxylic acid reductase, *chxJ*. In addition to the three strains described above, cycloheximide-like gene clusters were identified from seven additional *Streptomyces* and one *Saccharopolyspora* genome and were included in our dataset (**Supplementary Table S6**). In each case other than 2AW, the cycloheximide-like gene clusters have the tailoring gene cluster adjacent to the core PKS genes, as in the arrangement in YIM56141. One new gene arrangement was found in the cycloheximide-like gene clusters from *Streptomyces* sp. 769, *Streptomyces noursei* ATCC11455, and *Saccharopolyspora flava* DSM44771, where *chxF* is not between the PKS genes and *chxG.* In strains 769 and ATCC11455, *chxF* is downstream of the glutarimide core gene cluster instead of a putative SARP transcriptional regulator present in some of the glutarimide gene clusters. In addition, the final domain in the PKS of strains 769, ATCC11544, and DSM44711 is a condensation domain (IPR001242) instead of the traditional thioesterase domain (IPR029802) present in the other cycloheximide PKSs.

**FIGURE 4:**
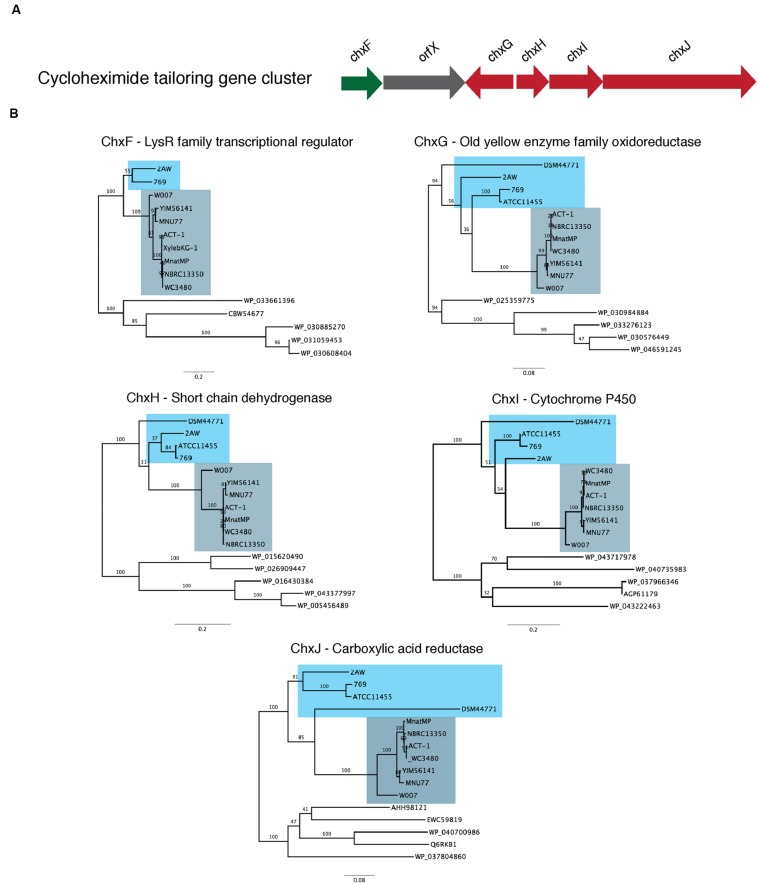
**Phylogenetic analysis of the proteins present in the cycloheximide tailoring gene cluster.** **(A)** Schematic representation of the cycloheximide tailoring gene cluster organization. **(B)** Maximum likelihood phylogenetic tree estimated of the protein sequence of each tailoring gene cluster.

We conducted a maximum-likelihood analysis of the proteins encoded by *chxF*, *chxG*, *chxH*, *chxI*, and *chxJ*. For all five of these proteins, orthologs from strains 2AW, 769, ATCC11455, and DSM44771 are in the basal portion of the tree relative to the others (**Figure 4**), suggesting they may reflect more ancestral lineages. However, there is not enough resolution to clarify the relationship between these ancestral clusters within the well-defined clade to which YIM56141 and NBRC13350 belong.

**FIGURE 5:**
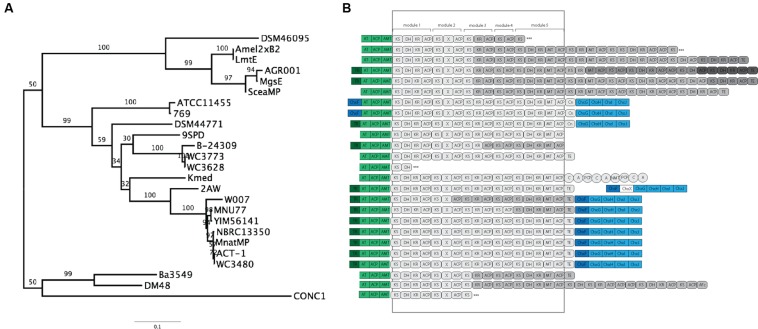
**Phylogenetic analysis and comparison of diverse glutaramide gene clusters.** **(A)** Maximum likelihood phylogenetic tree estimated of the protein sequence of the first ketosynthase domain in the PKS of diverse glutaramide gene clusters. **(B)** Schematic representation of glutarimide gene clusters. In green, loading module: ACP, acyl carrier protein; AT, malonyl-CoA ACP transacylase; AMT, amidotransferase. In gray: PKS, AT-less modular type I polyketide synthase; Gray-scale represented different genes to codify the multi-protein complex. Rounded rectangle, PKS domains: KS, ketosynthase; DH, dehydratase; ER, enoylreductase; MT, methyltransferase; ACP, acyl carrier protein; X, uncharacterized cyclization domain; TE, thiosesterase; Cn, condensation. Circles, NRPS domains: C, condensation; A, adenylation; PCP, thiolation and peptide carrier protein; NMT, methylation. In blue, cycloheximide gene cluster; dark blue transcriptional regulator, light blue tailoring. ∗∗∗ Partial PKS.

To further clarify the relationship between 2AW and the other ancestral lineages, we attempted to reconstruct the phylogeny of the core PKS gene cluster. The five modules that constitute the cycloheximide PKS and cycloheximide-like PKS are conserved in the biosynthesis of other glutarimide antibiotics such as 9-methylstreptimidone, lactimidomycin and iso-migrastatin, and in eight other glutaramide-like gene clusters found in Actinomycetes and *Burkholderia* spp. genomes (**Figure 5B**; **Supplementary Table S6**), even though only the first two PKS modules are thought to be needed to generate the glutaramide ring (**Figure 2A**). This observation suggests that the glutarimide biosynthesis family may have evolved from a basic five-module PKS.

The results above also suggested that the PKSs of the other glutaramide gene cluster might help to resolve the relationship between 2AW and the other cycloheximide-like gene clusters. The ketosynthase domain (KS) is commonly used to determine relationships between PKSs, because it is one of the essential domains present in the modules. We therefore conducted Maximum-likelihood analysis using the amino acid sequence of the first KS domain (KSI) as a representative of the entire PKS of all glutarimide gene clusters identified in public databases (**Figure 5A**), including three partial gene clusters (**Supplementary Table S6**). Lactimidomycin (LmtE) and iso-migrastatin (MgsE), which have similar gene clusters sharing additional modules in the PKSs, group together with the other four clusters that also share additional modules. These form a unique clade that diverged early from the other glutarimide-like clusters. The partial glutarimide-like gene cluster from an uncultured bacterial clone appears to be the most divergent among the gene clusters. For cycloheximide clusters, 2AW is next to and in a basal position to the other seven cycloheximide-like clusters, which grouped together in the previous phylogenetic trees. Meanwhile, 279, ATCC11455, and DSM44771 are located closer to other glutarimide clusters than to cycloheximide-specific clusters. Although there is not enough resolution to clarify the relationships between 279, ATCC11455, DSM44771, and the other glutaramide gene clusters, the phylogenetic analysis shows that the 2AW gene cluster is more closely related to the other cycloheximide gene clusters and may resemble an ancestral state in the evolution of the cycloheximide pathway. Phylogeny of an additional coding sequence of the AT gene showed a similar topology to that in the KSI tree (**Supplementary Figure S9**).

We postulate an evolutionary scenario in which the structural gene cluster and the tailoring gene cluster for cycloheximide production evolved independently of each other. The tailoring cluster accumulated genes that encode proteins with very diverse enzymatic activity. The 2AW tailoring gene cluster has an additional gene, designated here as *orfX*, which contains a condensation domain associated with non-ribosomal peptide synthetases. We hypothesize that this tailoring gene cluster evolved as a toolkit with a broad spectrum of activity, increasing the likelihood of interaction with other secondary metabolite gene clusters. Following its incorporation into a genome where it could interact with an ancestral glutarimide core gene cluster, the tailoring gene cluster lost genes not needed for activity, as we observe with *orfX*. The hypothesis of an independently evolved tailoring cluster may explain the different location of 279, ATCC11455, and DSM44771 in the KSI and AT tree relative to the other cycloheximide-like clusters. We hypothesize at least two independent events in which an ancestral tailoring gene cluster combined with an ancestral glutarimide core gene cluster. One such event produced a complete fusion of the two gene clusters where both gene clusters share the same transcriptional regulator, *chxF*, as in 279 and ATCC11455. Another such genetic event produced the fusion of the two gene clusters, maintaining within each cluster a transcriptional regulator, as in the YIM56141 gene cluster. Fusion of the two gene clusters then enabled the transfer and dissemination of a contiguous cycloheximide pathway cluster.

### Chemical Diversification in the Glutarimide Family

Diverse glutaramide antibiotics, such as isomigrastatin, lactimidomycin, streptimidone, and cyclohemiximide are known to inhibit eukaryotic translation. However, the potency of these antibiotics varies, and activity is correlated with the size of the moiety associated with the glutaramide ring (Obrig et al., 1971; Schneider-Poetsch et al., 2010). Crystal structures of 80S ribosomes from *Saccharomyces cerevisiae* in complex with lactimidomycin and cyclohemiximide indicate the location of the glutaramide ring in a pocket formed by universally conserved nucleotides of the 25S rRNA in the E-site. In addition to the hydrogen bonds formed between the 25S rRNA and the glutaramide ring, there are additional bonds with the succeeding hydroxyl and ketone group made in the first five modules of the PKSs (Garreau de Loubresse et al., 2014; **Figures 2C–F**). This structural observation could explain the conservation of the same five modules identified in all PKSs of the glutaramide family because the additional bonds create a strong interaction between the antibiotic and the ribosome. The interaction may have led to selection of the additional moieties, leading to highly diverse active compounds, since the glutarimide ring is necessary, but not sufficient, for activity (Schneider-Poetsch et al., 2010).

We propose an ancestral glutarimide core gene cluster formed from an ACP, a malonyl-CoA transacylase, an amidotransferase, and an AT-less PKS containing the five conserved modules. All glutaramide antibiotics evolved from this ancestral core gene cluster through a variety of strategies that resulted in the modification of the accessory moiety attached to the glutarimide ring. Phylogenetic analyses of the KSI and AT suggest an early evolution of the core gene cluster through the addition of five modules in the PKS, followed by loss-of-function mutations and duplication of the internal domain, thereby leading to pathways for the production of lactimidomycin and iso-migrastatin (**Figures 2E,F**). Interestingly, we identified a different strategy for increasing modules in the core gene cluster in the genome of *Kitasatospora mediocidica*, in which we identified a non-ribosomal peptide synthetase after the PKS, suggesting a hybrid PKS–NRPS. Another strategy to increase the chemical diversity of a basic glutarimide precursor may have been to maintain the same number of modules but to use a module iteratively, as in 9-methylstreptimidone (**Figure 2D**), or through the use of a tailoring gene cluster to modify a glutarimide precursor in trans, as in the case of cycloheximide (**Figure 2C**). The use of tailoring gene clusters to modify a scaffold is a common strategy to increase chemical diversity, and the glycopeptide family of antibiotics is just one example of how diverse modifications of the core heptapeptide scaffold produced by NRPSs can increase chemical diversity (Yim et al., 2014). Additional genes or gene clusters next to the NRPSs generate different modifications, such as glycosylation, acylation, chlorination, sulfonation, and methylation. However, the use of diverse tailoring gene clusters can create unique molecules too, such as in the example of simocyclinone, which is the assemblage of four different moieties, angucycline, dTDP-D-olivose, aminocoumarin and a linear polyene, produced by the fusion of four gene clusters (Trefzer et al., 2002).

## Conclusion

We identified three different antibiotics from *Streptomyces* sp. 2AW, an Alaskan soil isolate. Through comparative analysis of the *Streptomyces* sp. 2AW genome with other antibiotic-producing strains, we identified the antibiotic biosynthetic gene clusters and additional putative secondary metabolite gene clusters. These putative gene clusters likely produce metabolites we have not yet detected but which could be of scientific or clinical use. Unexpectedly, the cycloheximide gene cluster from our strain differed significantly from those previously identified. Analysis of this secondary metabolite gene cluster by sequence comparison defined several cycloheximide-like gene clusters as ‘other glutaramide-like gene clusters’ in public databases. Comparisons of the different gene clusters coupled with phylogenetic analysis of key proteins enabled us to propose a model in which cycloheximide biosynthetic genes evolved from an ancestral glutaramide core gene cluster through the addition of a tailoring gene cluster, with 2AW representing an intermediate step in pathway organization. Our results highlight the use and power of modern sequencing technologies and bioinformatics to advance our understanding of *Streptomyces* metabolite biosynthesis. Altogether, this work demonstrates how research in understudied natural environments, natural products chemistry, and bioinformatics can be combined to identify antibiotics and better understand the evolution of secondary metabolite biosynthetic pathways.

## Author Contributions

CM isolated *Streptomyces* sp. 2AW and screened it for antibacterial activity. EB made the initial observation of the anti-*Pythium* activity of *Streptomyces* sp. 2AW. ES, JM, GL, and GP contributed the isolation and purification of the three antibiotics. JM, EB, GP, and JR elucidated the structures of the antibiotics. CH participated in generating bioinformatics data. ES and GL were responsible for the generation, analysis, and interpretation of the bioinformatic data. HP and MT participated in interpretation of bioinformatic data. ES, GL, JM, AP, NB, ES, MT, and JH participated in the design of the experiments, interpretation of results, and drafted the manuscript. All authors read and approved the final manuscript.

## Conflict of Interest Statement

The authors declare that the research was conducted in the absence of any commercial or financial relationships that could be construed as a potential conflict of interest.

## Footnotes

1 http://medicine.yale.edu/keck/ycga/index.aspx

2 http://www.454.com/products/analysis-software/

3 http://tree.bio.ed.ac.uk/software/figtree/

4 http://genome.jgi.doe.gov

## Supplementary Material

FIGURE S1 **Hygromycin X PROTON**.

FIGURE S2 **Hygromycin X COSY**.

FIGURE S3 **Hygromycin X HSQC**.

FIGURE S4 **Hygromycin X HMBC**.

FIGURE S5 **Antibiotic structures.** Structures and molecular masses of **(A)** cycloheximide, **(B)** neutramycin and closed neutral macrolides, **(C)** hygromycin A and related hygromycin A molecules.

FIGURE S6 ***Streptomyces* sp. 2AW pyrimidine biosynthesis pathway. (A)** Schematic representation of the pyrimidine biosynthethic pathway. **(B)** *Streptomyces* sp. 2AW genes to codify the corresponding step in the pyrimidine biosynthesis.

FIGURE S7 **Schematic representation of the *Streptomyces* sp. 2AW genome**. All genes are color-coded, reverse and forward strand. The inside circle represents the location of the putative secondary metabolite gene clusters, color-coded by type. The scale is numbered in megabases.

FIGURE S8 **Comparisons of neutramycin and hygromycin A biosynthetic clusters to known clusters of related metabolites. (A)** Putative hygromycin A gene cluster compared to S. *hygroscopicu*s NRRL 238826, and **(B)** putative neutramycin gene cluster compared to chalcomycin and dihydrochalcomycin clusters in S. *bikiniensis* and *Streptomyces* sp. KCTC 0041 BP, respectively.

FIGURE S9 **Phylogenetic analysis of the malonyl-CoA ACP transacylase protein present in glutaramide gene cluster.** Maximum likelihood phylogenetic tree estimated of the AT protein sequence associated with glutaramide AT-less modular type I PKS as AT associates with others AT-less modular type I PKSs.

TABLE S1 **Nuclear magnetic resonance (NMR) spectral data for cycloheximide**.

TABLE S2 **Nuclear magnetic resonance spectral data for neutramycin**.

TABLE S3 **Nuclear magnetic resonance spectral data for hygromycin A**.

TABLE S4 ***Streptomyces* sp. 2AW genome basic characteristics**.

TABLE S5 **Gene annotation *Streptomyces* sp. 2AW genome**.

TABLE S6 **Glutarimide gene clusters found in public databases**.
