# Supplementary figures and images for: Genomic and Secondary Metabolite Analyses of Streptomyces sp. 2AW Provide Insight into the Evolution of the Cycloheximide Pathway
Source: Front Microbiol. 2016 May 3;7:573. doi: 10.3389/fmicb.2016.00573 (PMC4853412; doi:10.3389/fmicb.2016.00573)

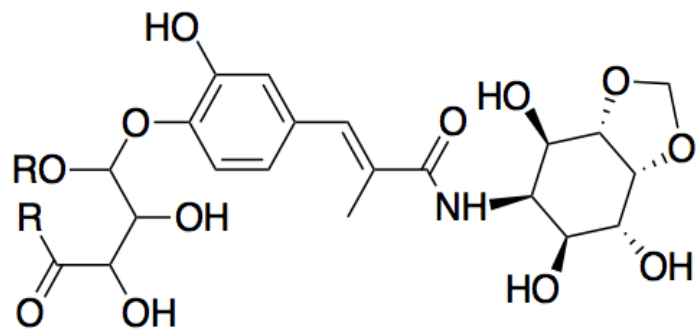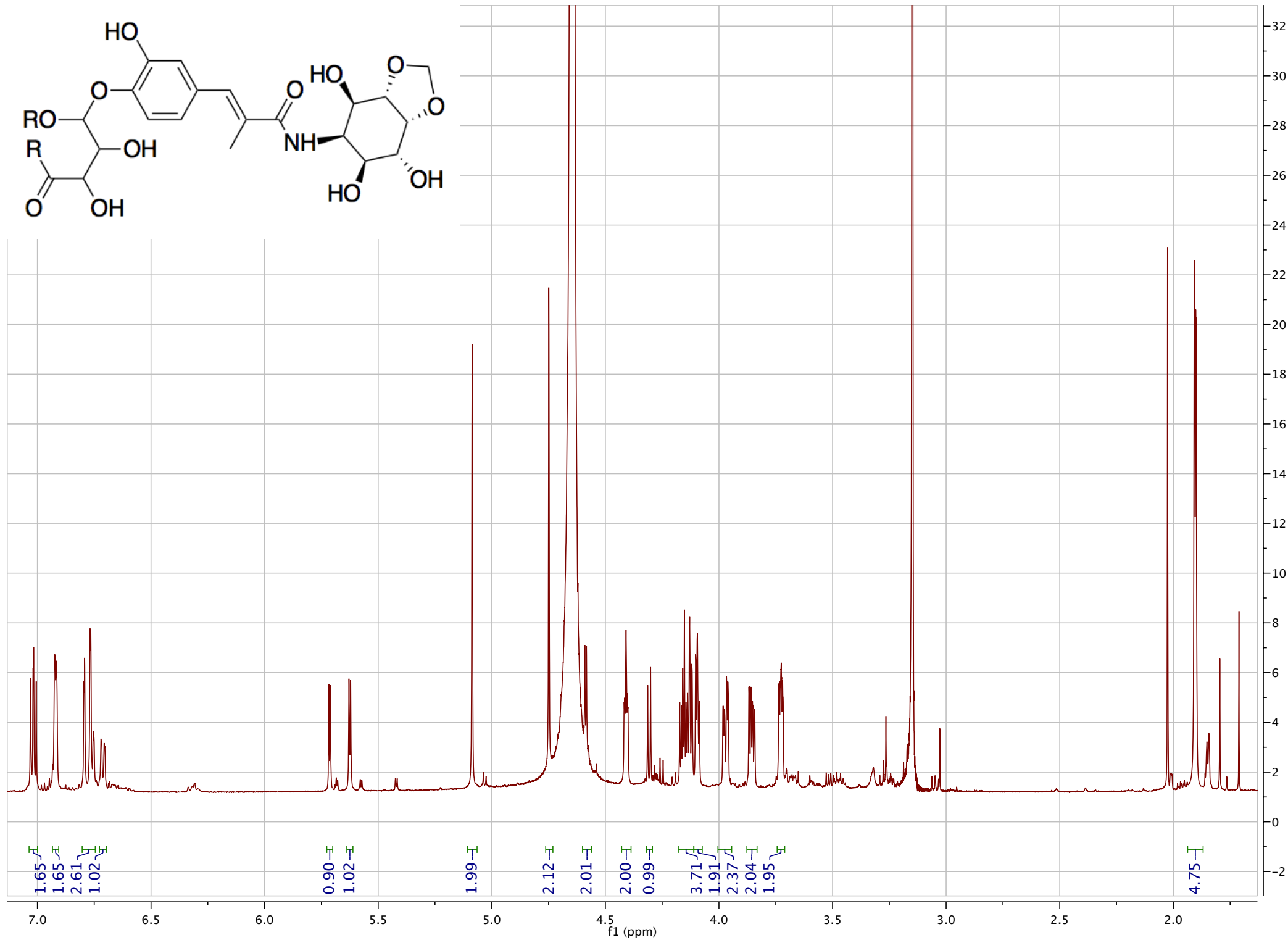

Supplement: FIGURE S1 — Hygromycin X PROTON. [file Image_1.PDF]

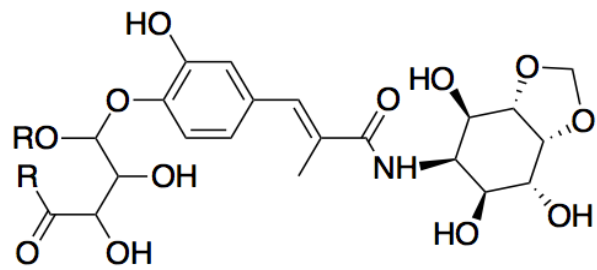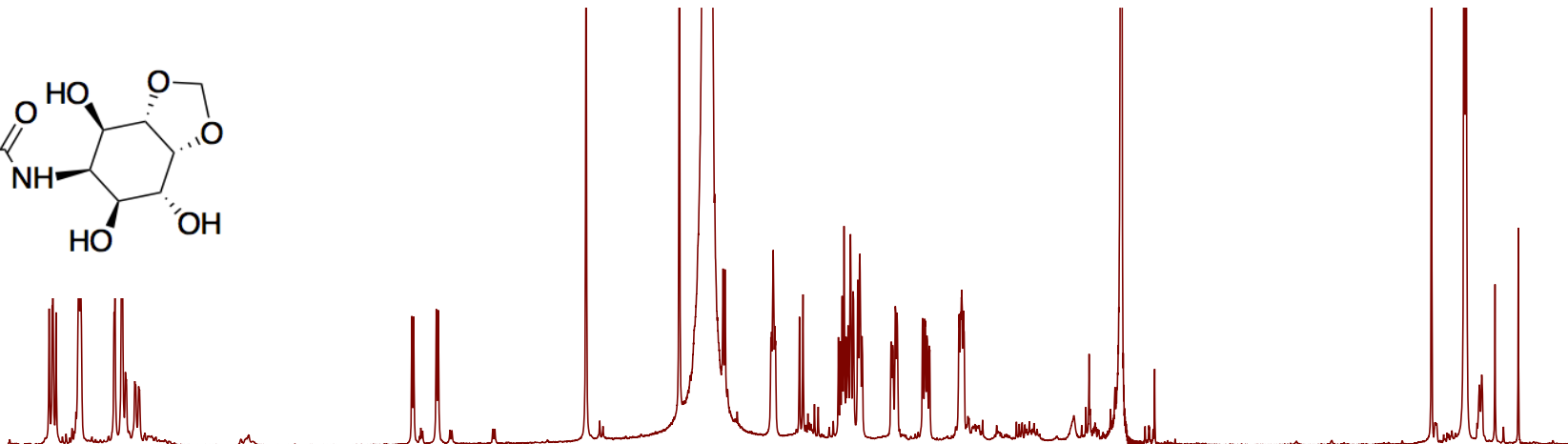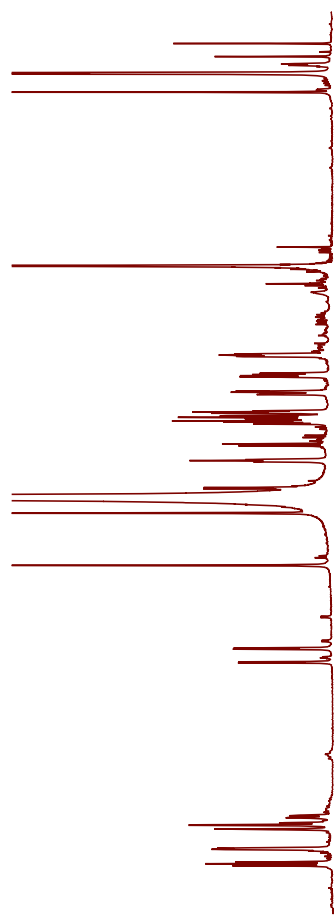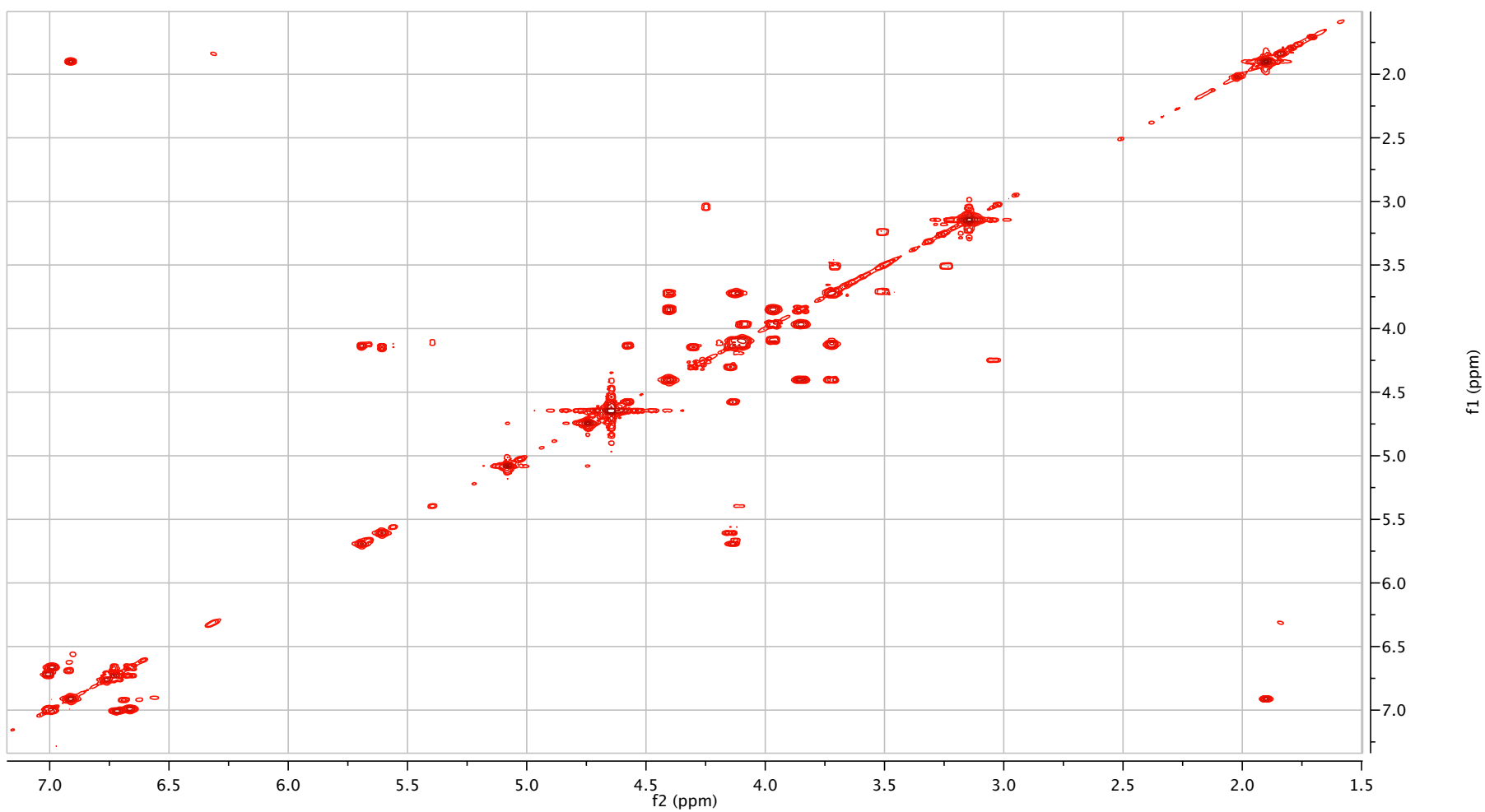

Supplement: FIGURE S2 — Hygromycin X COSY. [file Image_2.PDF]

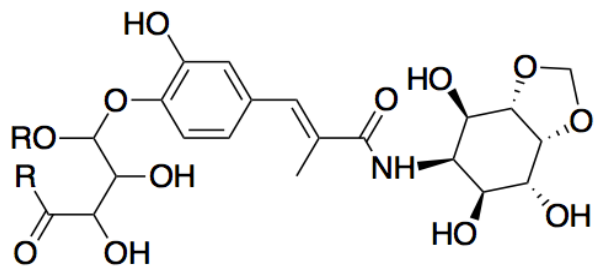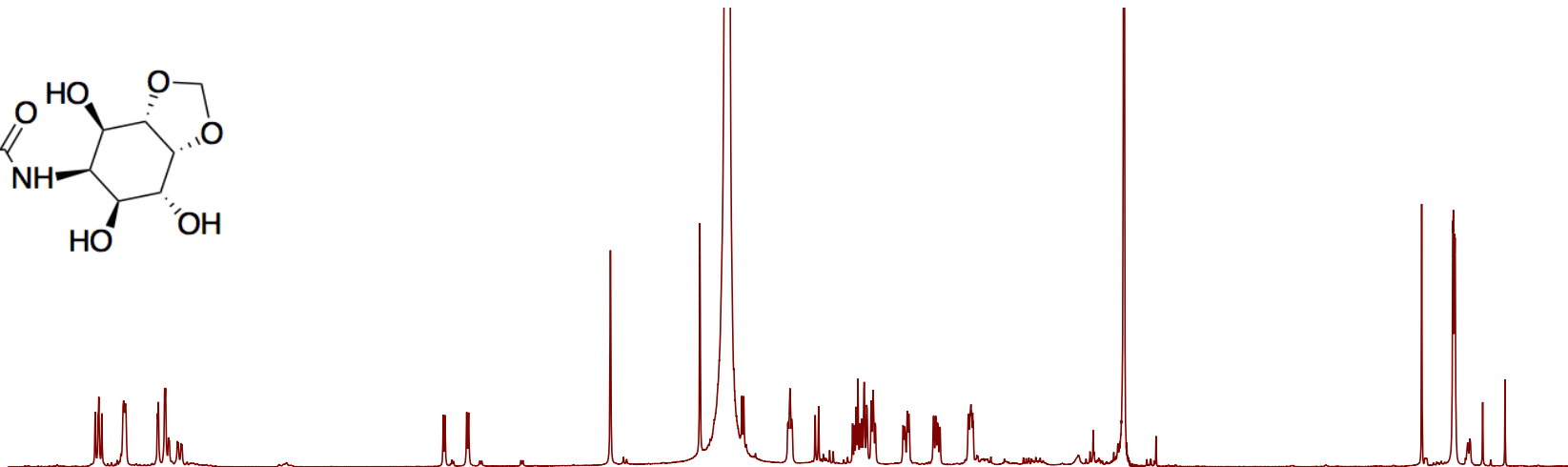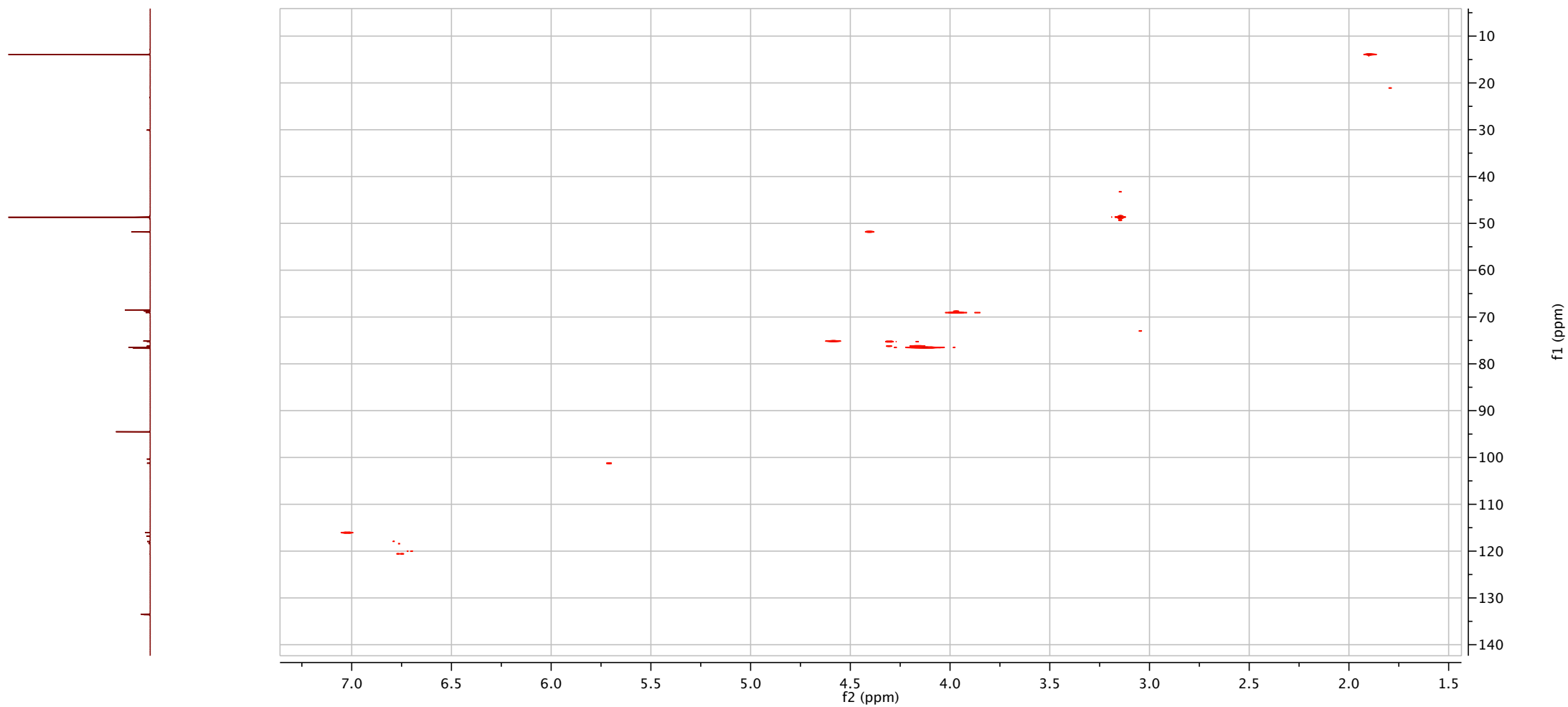

Supplement: FIGURE S3 — Hygromycin X HSQC. [file Image_3.PDF]

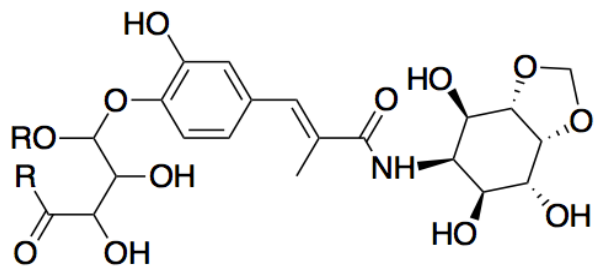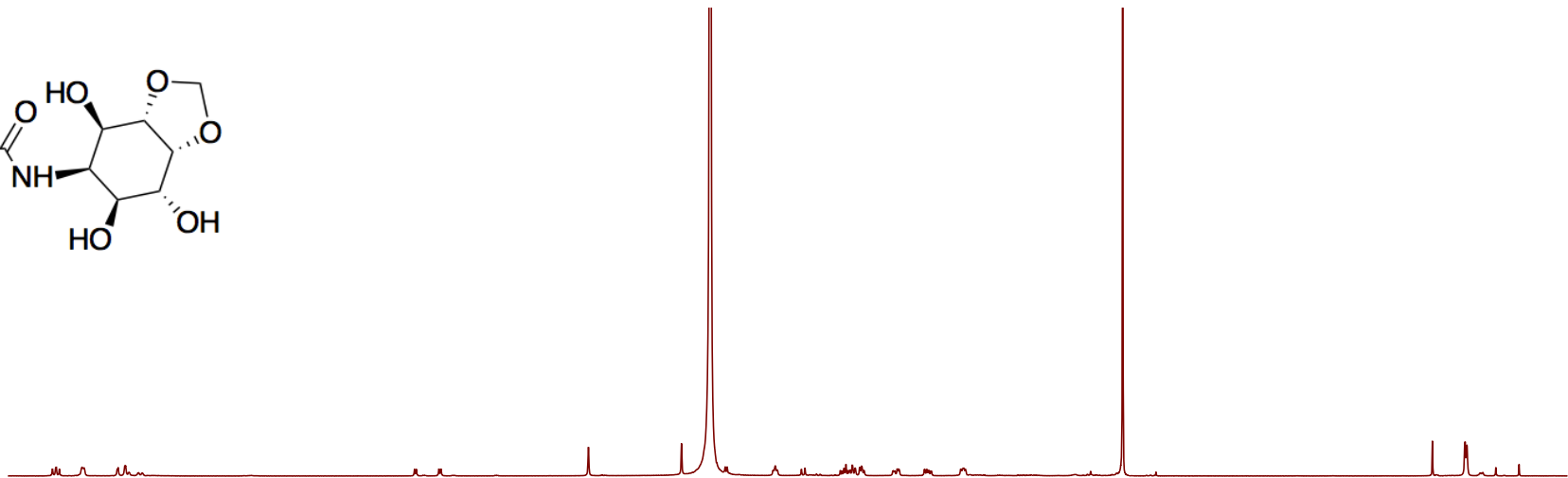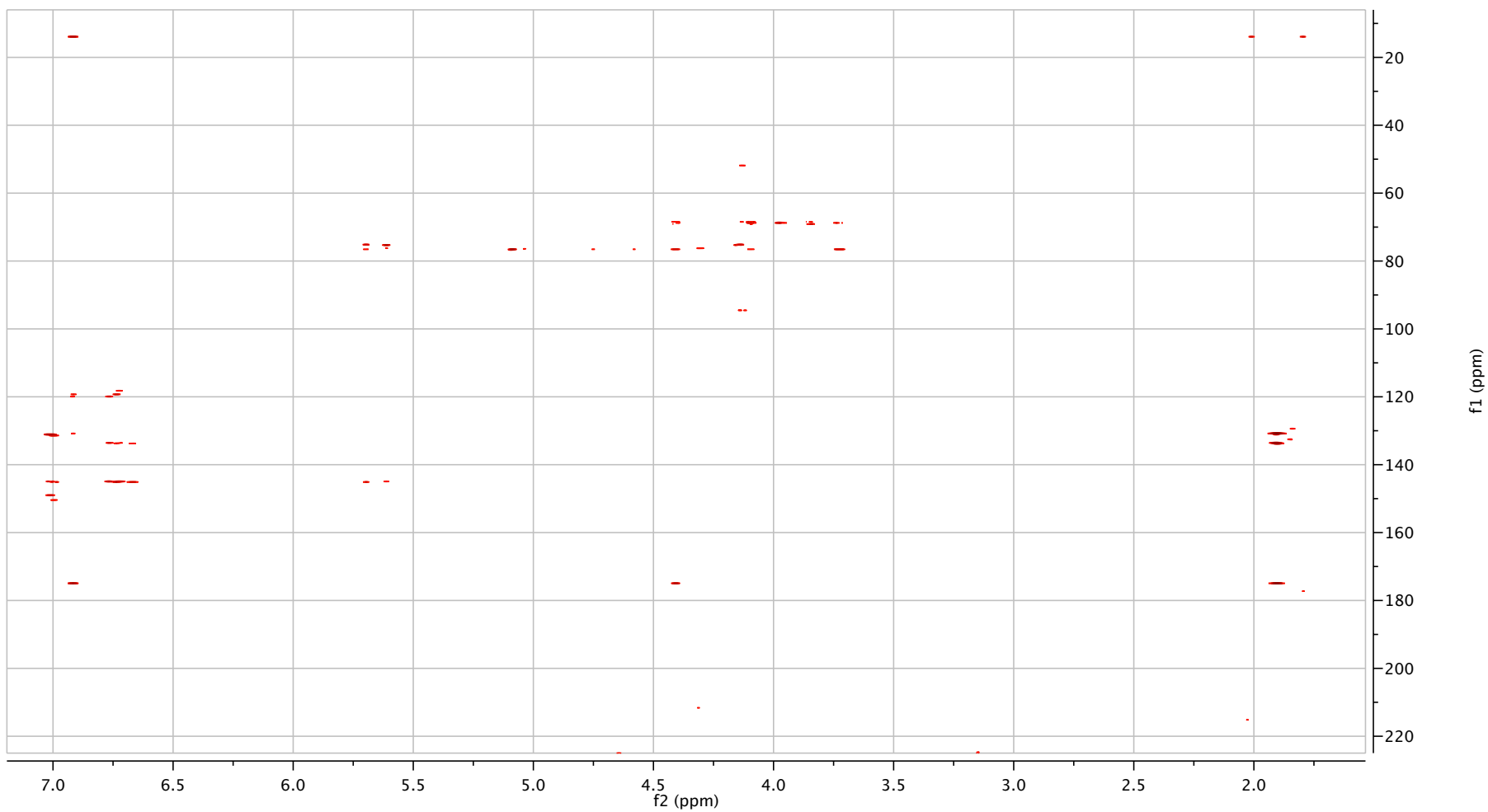

Supplement: FIGURE S4 — Hygromycin X HMBC. [file Image_4.PDF]

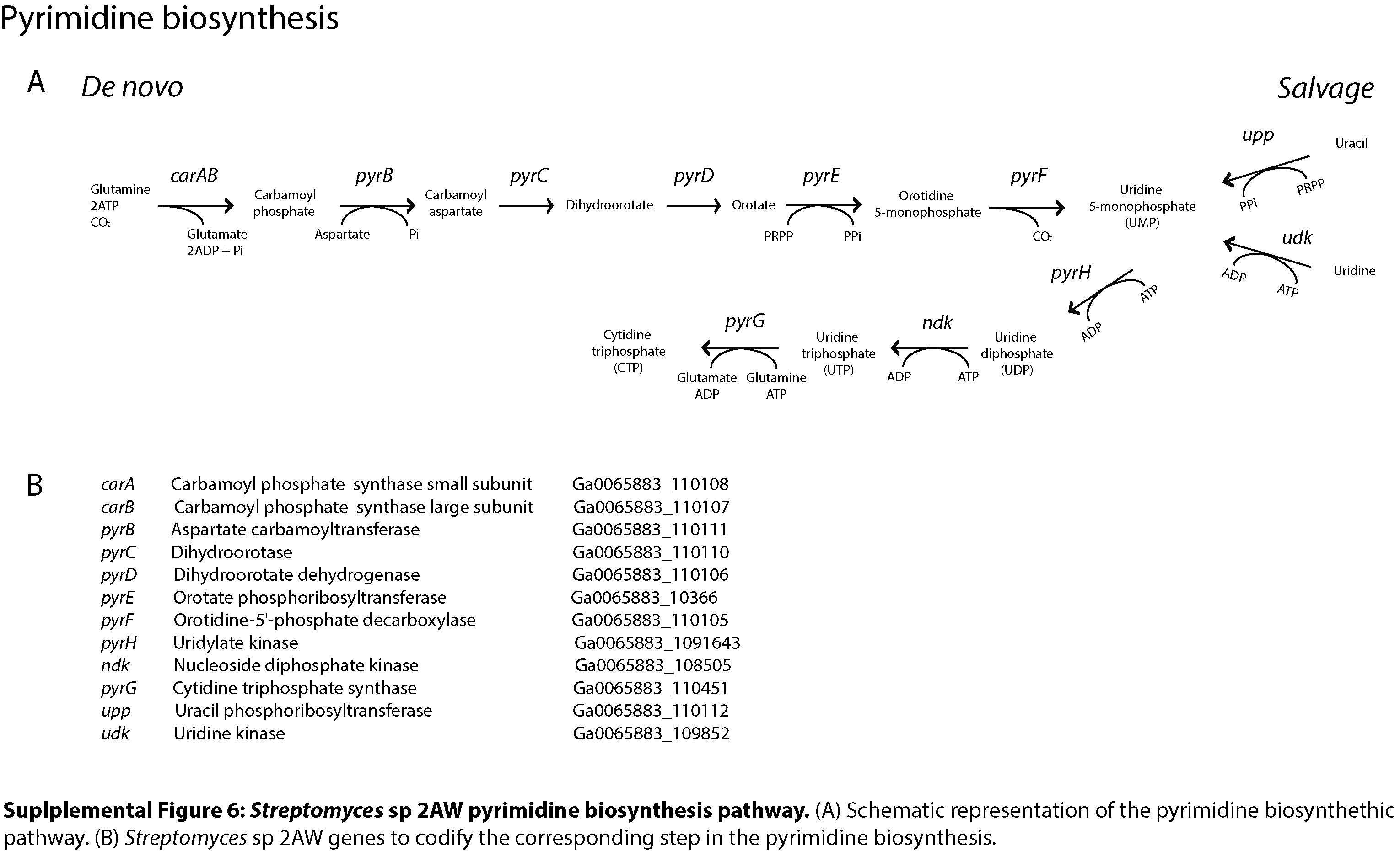

Supplement: FIGURE S6 — Streptomyces sp. 2AW pyrimidine biosynthesis pathway. (A) Schematic representation of the pyrimidine biosynthethic pathway. (B) Streptomyces sp. 2AW genes to codify the corresponding step in the pyrimidine biosynthesis. [file Image_6.TIF]

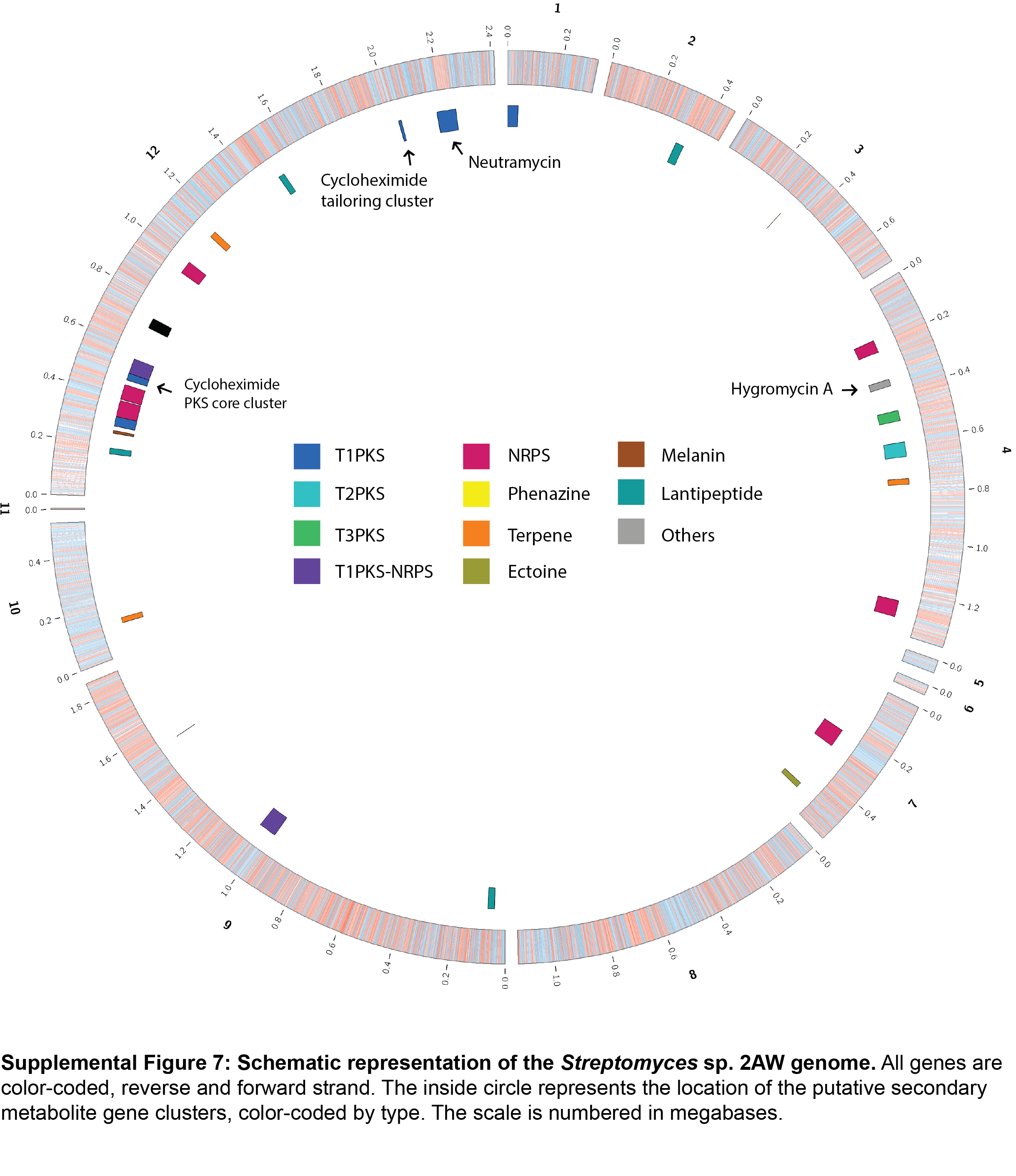

Supplement: FIGURE S7 — Schematic representation of the Streptomyces sp. 2AW genome. All genes are color-coded, reverse and forward strand. The inside circle represents the location of the putative secondary metabolite gene clusters, color-coded by type. The scale is numbered in megabases. [file Image_7.TIF]

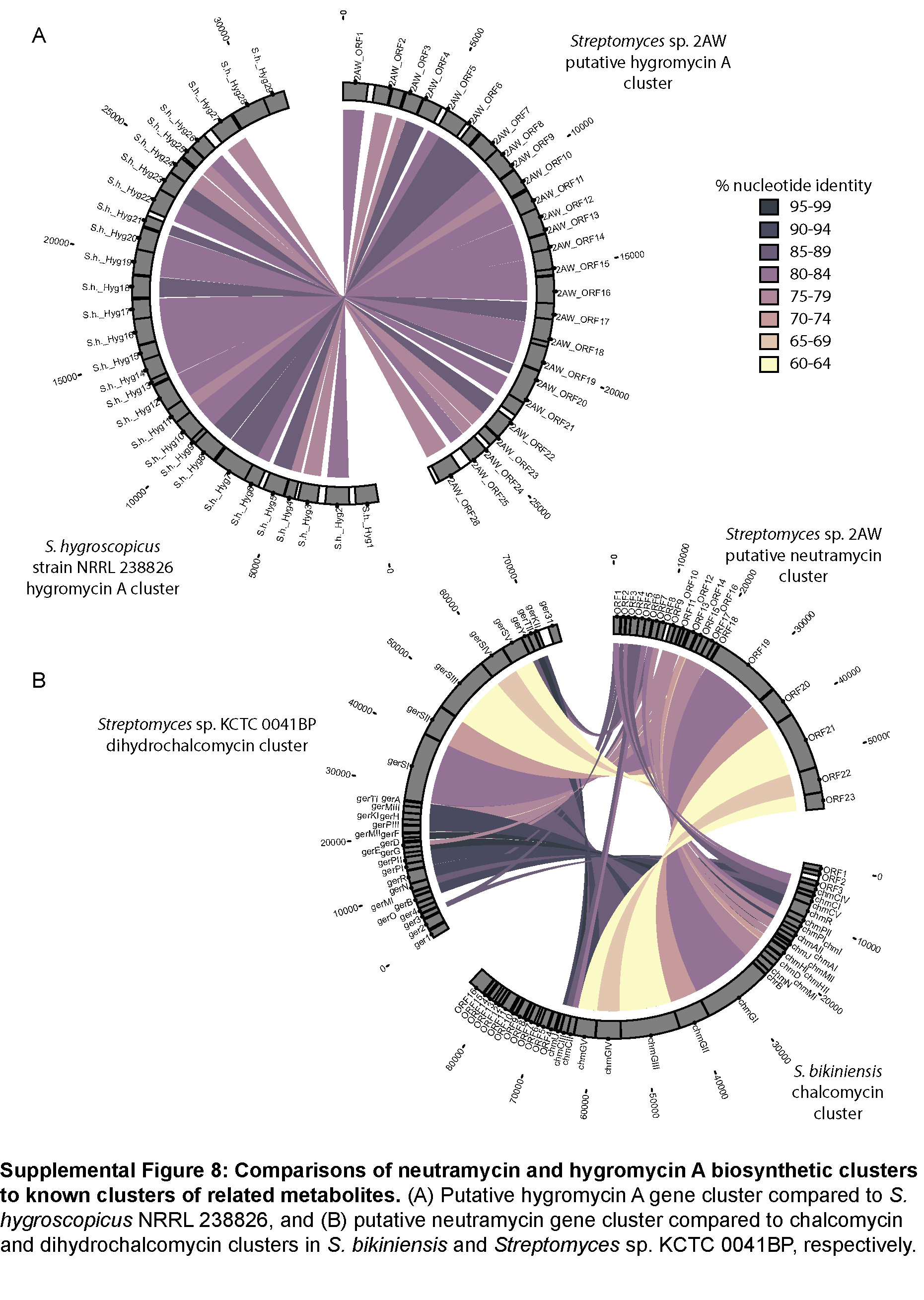

Supplement: FIGURE S8 — Comparisons of neutramycin and hygromycin A biosynthetic clusters to known clusters of related metabolites. (A) Putative hygromycin A gene cluster compared to S. hygroscopicus NRRL 238826, and (B) putative neutramycin gene cluster compared to chalcomycin and dihydrochalcomycin clusters in S. bikiniensis and Streptomyces sp. KCTC 0041 BP, respectively. [file Image_8.TIF]

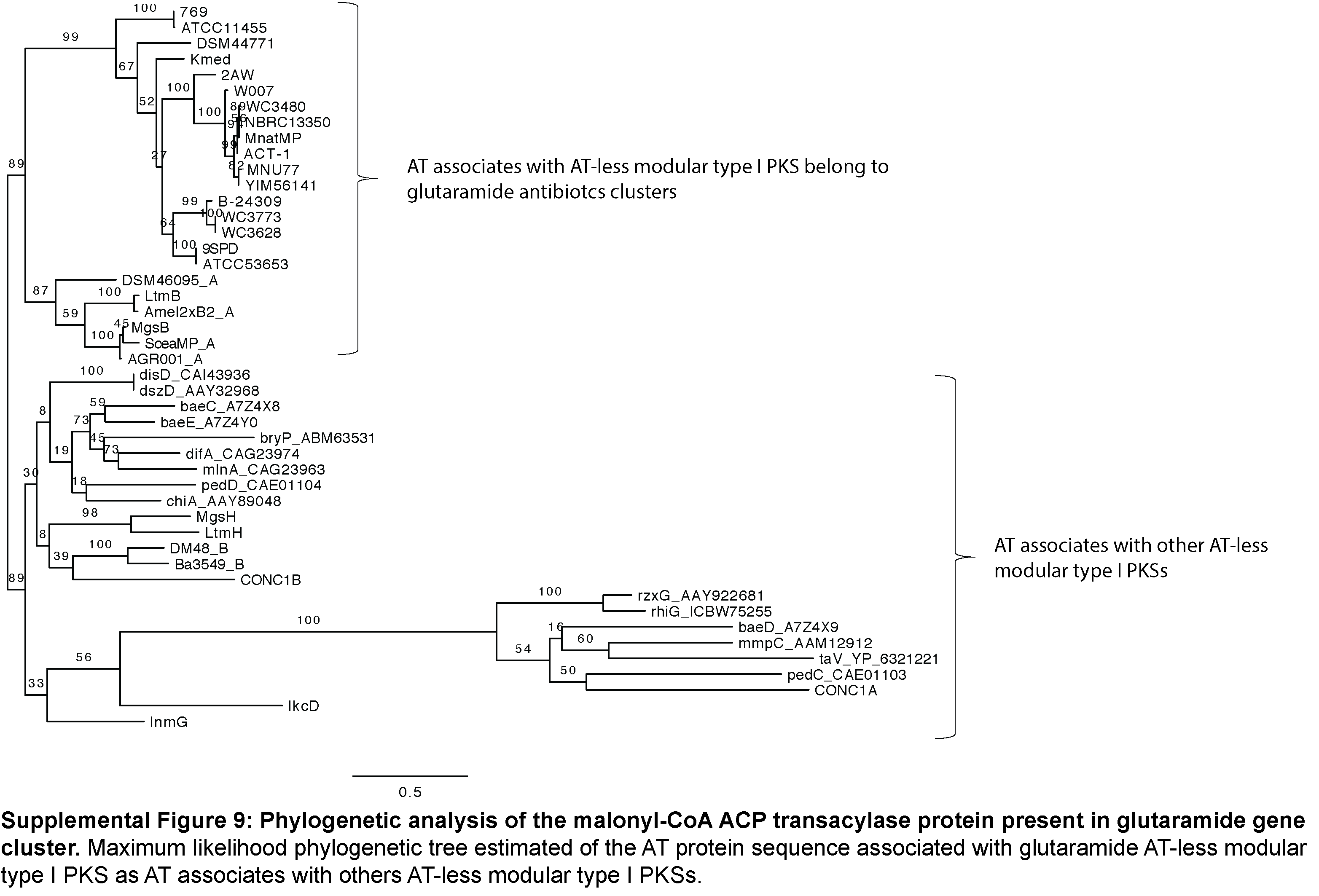

Supplement: FIGURE S9 — Phylogenetic analysis of the malonyl-CoA ACP transacylase protein present in glutaramide gene cluster. Maximum likelihood phylogenetic tree estimated of the AT protein sequence associated with glutaramide AT-less modular type I PKS as AT associates with others AT-less modular type I PKSs. [file Image_9.TIF]
