# Supplementary material for: Genomic and Secondary Metabolite Analyses of Streptomyces sp. 2AW Provide Insight into the Evolution of the Cycloheximide Pathway
Source: Front Microbiol. 2016 May 3;7:573. doi: 10.3389/fmicb.2016.00573 (PMC4853412; doi:10.3389/fmicb.2016.00573)
Supplement: FIGURE S5 — Antibiotic structures. Structures and molecular masses of (A) cycloheximide, (B) neutramycin and closed neutral macrolides, (C) hygromycin A and related hygromycin A molecules. [file Image_5.PDF]

A

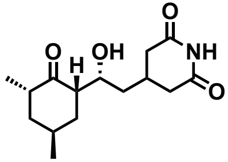

Cycloheximide
m/z 304.1520
calcd for $C_{15}H_{23}NO_4$
$[M+Na]^+$ 304.1525

B

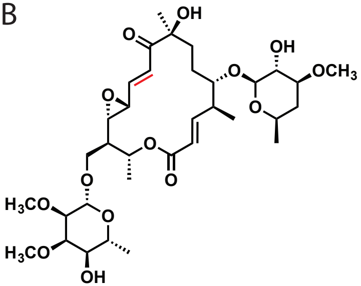

Neutramycin
m/z 709.3304
calcd for $C_{34}H_{54}O_{14}$
$[M+Na]^+$ 709.3411

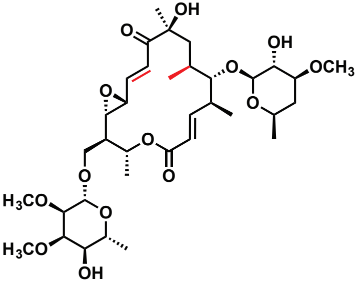

Chalcomycin

calcd for $C_{34}H_{56}O_{14}$
$[M+Na]^+$ 700.3670

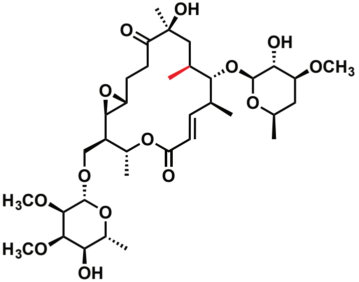

Dihydrochalcomycin

calcd for $C_{35}H_{58}O_{14}$
$[M+Na]^+$ 702.3827

C

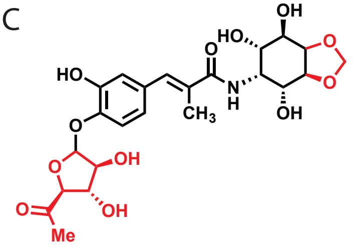

Hygromycin A
m/z 512.1759
calcd for $C_{23}H_{29}NO_{12}$
$[M+H]^+$ 512.1768

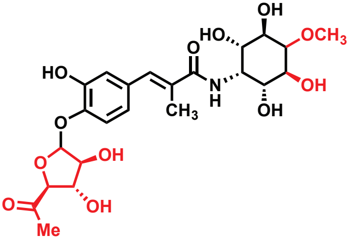

Methyoxyhygromycin A
m/z 514.1902
calcd for $C_{23}H_{32}NO_{12}$
$[M+H]^+$ 514.1925

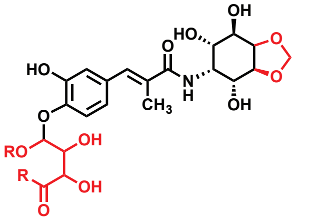

Proposed Streptomyces sp. 2AW
Hygromycin A Derivative
m/z 516.2057
possible formula: $C_{23}H_{34}NO_{12}$
$[M+H]^+$ 516.2081
