# Supplementary material for: Genomic and Secondary Metabolite Analyses of Streptomyces sp. 2AW Provide Insight into the Evolution of the Cycloheximide Pathway
Source: Front Microbiol. 2016 May 3;7:573. doi: 10.3389/fmicb.2016.00573 (PMC4853412; doi:10.3389/fmicb.2016.00573)
Supplement: TABLE S1 — Nuclear magnetic resonance (NMR) spectral data for cycloheximide. [file Table_1.DOCX]

| pos. | δ_H_, mult. (*J* in Hz) | δ_C_ |
| --- | --- | --- |
| N1 | 8.23, bs | - |
| 2 | - | 172.4 |
| 3 | \| 2.77, dd (4.1, 17.0) \| \| --- \| \| 2.43, m \| \| 2.32, m \| \| - \| | 38.5 |
| 4 | 2.43, m | 27.6 |
| 5 | 2.32, m | 37.2 |
| 6 | - | 172.2 |
| 7a | 1.20, m | 37.9 |
| 7b | 1.64, m |  |
| 8 | 4.20, dt (2.5, 10.9) | 66.6 |
| 9 | 2.50, m | 50.1 |
| 10 | - | 216.6 |
| 11 | 2.63, m | 40.6 |
| 12a | 1.62, m | 42.6 |
| 12b | 1.90, m |  |
| 13 | 2.21, m | 26.7 |
| 14a | 1.81, m | 33.0 |
| 14b | 1.94, m |  |
| 15 | 0.99, d (6.5) | 14.2 |
