# Supplementary material for: Genomic and Secondary Metabolite Analyses of Streptomyces sp. 2AW Provide Insight into the Evolution of the Cycloheximide Pathway
Source: Front Microbiol. 2016 May 3;7:573. doi: 10.3389/fmicb.2016.00573 (PMC4853412; doi:10.3389/fmicb.2016.00573)
Supplement: TABLE S2 — Nuclear magnetic resonance spectral data for neutramycin. [file Table_2.DOCX]

| pos. | δ_H_, mult. (*J* in Hz) | δ_C_ |
| --- | --- | --- |
| 1 | - | 167.2 |
| 2 | 6.03, d (15.5) | 122.0 |
| 3 | 6.57, dd (10.6, 15.8) | 152.5 |
| 4 | 2.66, m | 41.6 |
| 5 | 3.51, m | 82.7 |
| 6a | 0.73, m | 28.1 |
| 6b | 2.02, m |  |
| 7 | 1.96, m | 31.7 |
| 8 | - | 79.0 |
| 9 | - | 201.5 |
| 10 | 6.98, d (15.3) | 127.3 |
| 11 | 6.43, dd (9.4, 15.8) | 146.3 |
| 12 | 3.41, m | 59.9 |
| 13 | 3.07, dd (1.8, 9.3) | 60.0 |
| 14 | 1.41, s | 50.5 |
| 15 | 5.41, m | 69.6 |
| 16 | 1.35, d (6.4) | 18.3 |
| 17 | 1.16, d (6.6) | 18.3 |
| 18 | 1.41, s | 26.2 |
| 19 | 3.69, m | 67.9 |
|  | 4.12, dd (2.7, 10.3) |  |
| 1’ | 4.25, d (7.7) | 105.9 |
| 2’ | 3.16, m | 75.7 |
| 3’ | 3.79, m | 71.9 |
| 4’ | 1.21, m | 38.4 |
|  | 2.11, ddd (1.4, 5.0, 12.6) |  |
| 5’ | 3.58, m | 68.6 |
| 6’ | 1.22, d^a^ | 21.1 |
| 7’ | 3.41, s | 57.2 |
| 1’’ | 4.59, d (8.0) | 102.0 |
| 2’’ | 3.09, dd (2.7, 8.0) | 81.6 |
| 3’’ | 3.22, m | 81.6 |
| 4’’ | 3.18, m | 74.4 |
| 5’’ | 3.67, m | 70.8 |
| 6’’ | 1.24, d^a^ | 17.9 |
| 7’’ | 3.56, s | 59.3 |
| 8’’ | 3.57, s | 61.9 |

^a^These peaks overlapped and appeared to be a triplet.
