# Supplementary material for: Genomic and Secondary Metabolite Analyses of Streptomyces sp. 2AW Provide Insight into the Evolution of the Cycloheximide Pathway
Source: Front Microbiol. 2016 May 3;7:573. doi: 10.3389/fmicb.2016.00573 (PMC4853412; doi:10.3389/fmicb.2016.00573)
Supplement: TABLE S3 — Nuclear magnetic resonance spectral data for hygromycin A. [file Table_3.DOCX]

| pos. | anomer 1 | | anomer 2 | |
| --- | --- | --- | --- | --- |
|  | δ_H_, mult. (*J* in Hz) | δ_C_ | δ_H_, mult. (*J* in Hz) | δ_C_ |
| 1 | 3.85, ddd (1.4, 5.0, 9.5) | 68.9 | 3.85, ddd (1.4, 5.0, 9.5) | 68.9 |
| 2 | 4.41, ddd (1.7, 3.8, 5.2) | 51.8 | 4.41, ddd (1.7, 3.8, 5.2) | 51.8 |
| 3 | 3.73, ddd (1.6, 3.8, 6.4) | 68.7 | 3.73, ddd (1.6, 3.8, 6.4) | 68.7 |
| 4 | 4.13, m | 76.6 | 4.13, m | 76.6 |
| 5 | 4.09, ddd (1.1, 3.9, 5.4) | 76.7 | 4.09, ddd (1.1, 3.9, 5.4) | 76.7 |
| 6 | 3.97, ddd (1.4, 3.7, 9.5) | 69.2 | 3.97, ddd (1.4, 3.7, 9.5) | 69.2 |
| 7a | 4.75, s | 94.6 | 4.75, s | 94.6 |
| 7b | 5.09, s |  | 5.09, s |  |
| 1’ | - | 174.9 | - | 174.9 |
| 2’ | - | 131.0 | - | 131.0 |
| 3’ | 6.92, d (1.7) | 133.6 | 6.91, d (1.7) | 133.5 |
| 4’ | - | 131.5 | - | 131.2 |
| 5’ | 7.01, d (6.6 or 8.3) | 117.0 | 7.02, d (6.6 or 8.3) | 116.3 |
| 6’ | - | 147.8 | - | 149.2 |
| 7’ | - | 144.5 | - | 144.4 |
| 8’ | 6.71, dd (2, 8.3) | 120.0 | 6.76, dd (buried) | 120.6 |
| 9’ | 6.77, d (2.0) | 118.2 | 6.79, d (2.0) | 117.8 |
| 10’ | 1.90, d (1.9) | 14.0 | 1.90, d (1.9) | 14.0 |
| 1’’ | 5.71, d (3.9) | 101.3 | 5.63, d (4.4) | 100.5 |
| 2’’ | 4.15, m | 76.7 | 4.16, m | 76.3 |
| 3’’ | 4.58, d (4.3) | 75.3 | 4.31, d (7.7) | 75.4 |
| 4’’ | - | 210.0 | - | 211.5 |

|  |
| --- |

Comparison of hygromycin A (1) and hygromycin A derivative in MeOD

| hygromycin A  δ_H_, mult. (*J* in Hz) | hygromycin A derivative  δ_H_, mult. (*J* in Hz) |
| --- | --- |
| 7.28, br s | 7.26, br s |
| 7.24, d (8.5) | 7.24, dd (3.0, 8.7) |
| 6.92, s | 6.94, dd (2.0, 6.4) |
| 6.88, dd (8.5, 1.6) | 6.88, m |
| 5.64, d (4.1) | 5.63, d (4.3) |
| 5.26, s | 5.23, s |
| 4.82, s | 4.79, s |
| 4.53, dd (6.1, 7.2) | 4.50, dd (2.7, 6.3) |
| 4.37, dd (6.6, 6.6) | 4.35, d (7.2) |
| 4.29, d (6.3) | - |
| 4.27-4.14, m | 4.27-4.14, m |
| 4.00, dd (6.9, 6.9) | 3.96, dd (6.6, 6.6) |
| 3.83, dd (2.8, 2.8) | 3.80, dd (2.9, 2.90 |
| 2.15, s | 2.13, d (1.3) |
| 2.15, s | - |

**References:**

1. **Donohoe TJ**, **Flores A**, **Bataille CJR**, **Churruca F**. 2009. Synthesis of (−)-Hygromycin A: Application of Mitsunobu Glycosylation and Tethered Aminohydroxylation. Angew. Chem. Int. Ed. **48**:6507–6510.
