# Supplementary material for: Genomic and Secondary Metabolite Analyses of Streptomyces sp. 2AW Provide Insight into the Evolution of the Cycloheximide Pathway
Source: Front Microbiol. 2016 May 3;7:573. doi: 10.3389/fmicb.2016.00573 (PMC4853412; doi:10.3389/fmicb.2016.00573)
Supplement: TABLE S6 — Glutarimide gene clusters found in public databases. [file Table_6.DOCX]

**Supplemental Table 6 - Glutarimide gene clusters found in public databases**.

| Organism | Abbreviation | Gene Cluster Identifier | Glutaramide Type |
| --- | --- | --- | --- |
| *Streptomyces* sp. 2AW | 2AW | Ga0065883_112411-Ga0065883_112407 / Ga0065883_1121714 - Ga0065883 ^a^ | Cycloheximide |
| *Streptomyces* sp. YIM56141 | YIM56141 | AFO59862 - AFO59871 | Cycloheximide |
| *Streptomyces griseus* subsp. *griseus* NBRC 13350 | NBRC13350 | BAG23610 - BAG23619 | Cycloheximide |
| *Streptomyces* sp. W007 | Woo7 | WP_007451490 - WP_007451508 | Cycloheximide-like |
| Streptomyces griseus subspecies griseus WC3480 | WC3480 | P212_03591 - P212_03600 ^b^ | Cycloheximide-like |
| *Streptomyces* sp. ACT-1 | ACT-1 | WP_003971101 - WP_003971111 | Cycloheximide-like |
| *Streptomyces* sp. MNU77 | MNU77 | KIZ26038 - KIZ26047 | Cycloheximide-like |
| *Streptomyce*s sp. MnatMP-M77 | MnatMP | YW3DRAFT_03469 - YW3DRAFT_03478 ^a^ | Cycloheximide-like |
| *Streptomyces* sp. 769 | 769 | WP_039638735 - WP_039638744 | Cycloheximide-like |
| *Streptomyces noursei* ATCC 11455 | ATCC11455 | S._noursei_presub_7394 - S.noursei_presub_7401 ^a^ | Cycloheximide-like |
| *Saccharopolyspora flava* DSM 44771 | DSM44771 | Ga0056047_04090 - Ga0056047_04098 ^a^ | Cycloheximide-like |
| *Streptomyces himastatinicus* ATCC 53653 | 9SPD | CCC21119 - CCC21125 | 9-methylstreptimidone |
| *Streptomyces monomycini* | B-24309 | WP_030019158 - WP_030019163 | 9-methylstreptimidone-like |
| *Streptomyces* sp. NRRL WC-3773 | WC3773 | WP_031001628 - WP_031001625 | 9-methylstreptimidone-like |
| *Streptomyces thermotolerans* strain NRRL WC-3628 | WC3628 | P228_05703 - P228_05706 ^b^ | 9-methylstreptimidone-like |
| *Streptomyces amphibiosporus* ATCC 53964 | LTM | ACY01397 - ACY01405 | Lactimidomycin |
| *Streptomyces* sp. Amel2xB2 | Amel2xB2 | K378DRAFT_02575 - K378DRAFT_02579 ^a^ | Lactimidomycin-like |
| *Streptomyces platensis* NRRL 18993 | MGS | ACY01386 - ACY01395 | iso-migrastatin |
| *Streptomyces auratus* AGR0001 | AGR001 | WP_006606836 - WP_006606847 | iso-migrastatin-like |
| *Streptomyces* sp. SceaMP-e96 | SceaMP | YWIDRAFT_03980 - YWIDRAFT03984 | iso-migrastatin-like |
| *Amycolatopsis rifamycinica* strain DSM 46095 | DSM46095 | DV20_12310 - DV20_12325 ^a^ | Unknown |
| *Kitasatospora mediocidica* | Kmed | BS80DRAFT_02834 - BS80DRAFT_02838 ^a^ | Unknown |
| Uncultured bacterium clone CONC1 | CONC1 | KJ604852 | Unknown |
| *Burkholderia mallei* strain gladioli DM48 | DM48 | KGC12726 - KGC12967 | Unknown |
| *Burkholderia andropogonis* Ba3549 | Ba3549 | W813DRAFT_05119 - W813DRAFT05123 ^a^ | Unknown |
| ^a^ JGI Genome Portal (http://genome.jgi.doe.gov) | | | |
| ^b^ Natural Products Genomics (http://www.igb.illinois.edu/labs/metcalf/gcf/index.html) | | | |
